# Mutual influence of selenium nanoparticles and FGF2-STAB^®^ on biocompatible properties of collagen/chitosan 3D scaffolds: in vitro and ex ovo evaluation

**DOI:** 10.1186/s12951-021-00849-w

**Published:** 2021-04-13

**Authors:** Johana Muchová, Vanessa Hearnden, Lenka Michlovská, Lucie Vištejnová, Anna Zavaďáková, Kristýna Šmerková, Silvia Kočiová, Vojtěch Adam, Pavel Kopel, Lucy Vojtová

**Affiliations:** 1 CEITEC–Central European Institute of Technology, Brno University of Technology, Purkynova 656/123, 612 00 Brno, Czech Republic; 2 Department of Materials Science and Engineering, Kroto Research Institute, North Campus, University of Sheffield, Broad Lane, Sheffield, S3 7HQ UK; 3 Biomedical Center, Medical Faculty in Pilsen, Charles University, Alej Svobody 1655/76, 323 00 Pilsen, Czech Republic; 4 Department of Chemistry and Biochemistry, Mendel University in Brno, Zemedelska 1665/1, 613 00 Brno, Czech Republic; 5 Department of Inorganic Chemistry, Faculty of Science, Palacky University, 17. Listopadu 12, 771 46 Olomouc, Czech Republic

**Keywords:** Selenium nanoparticles, Collagen, Chitosan, Fibroblast growth factor 2, 3D porous scaffold

## Abstract

In a biological system, nanoparticles (NPs) may interact with biomolecules. Specifically, the adsorption of proteins on the nanoparticle surface may influence both the nanoparticles' and proteins' overall bio-reactivity. Nevertheless, our knowledge of the biocompatibility and risk of exposure to nanomaterials is limited. Here, in vitro and ex ovo biocompatibility of naturally based crosslinked freeze-dried 3D porous collagen/chitosan scaffolds, modified with thermostable fibroblast growth factor 2 (FGF2-STAB^®^), to enhance healing and selenium nanoparticles (SeNPs) to provide antibacterial activity, were evaluated. Biocompatibility and cytotoxicity were tested in vitro using normal human dermal fibroblasts (NHDF) with scaffolds and SeNPs and FGF2-STAB^®^ solutions. Metabolic activity assays indicated an antagonistic effect of SeNPs and FGF2-STAB^®^ at high concentrations of SeNPs. The half-maximal inhibitory concentration (IC50) of SeNPs for NHDF was 18.9 µg/ml and IC80 was 5.6 µg/ml. The angiogenic properties of the scaffolds were monitored ex ovo using a chick chorioallantoic membrane (CAM) assay and the cytotoxicity of SeNPs over IC80 value was confirmed. Furthermore, the positive effect of FGF2-STAB^®^ at very low concentrations (0.01 µg/ml) on NHDF metabolic activity was observed. Based on detailed in vitro testing, the optimal concentrations of additives in the scaffolds were determined, specifically 1 µg/ml of FGF2-STAB^®^ and 1 µg/ml of SeNPs. The scaffolds were further subjected to antimicrobial tests, where an increase in selenium concentration in the collagen/chitosan scaffolds increased the antibacterial activity. This work highlights the antimicrobial ability and biocompatibility of newly developed crosslinked collagen/chitosan scaffolds involving FGF2-STAB^®^ and SeNPs. Moreover, we suggest that these sponges could be used as scaffolds for growing cells in systems with low mechanical loading in tissue engineering, especially in dermis replacement, where neovascularization is a crucial parameter for successful skin regeneration. Due to their antimicrobial properties, these scaffolds are also highly promising for tissue replacement requiring the prevention of infection.
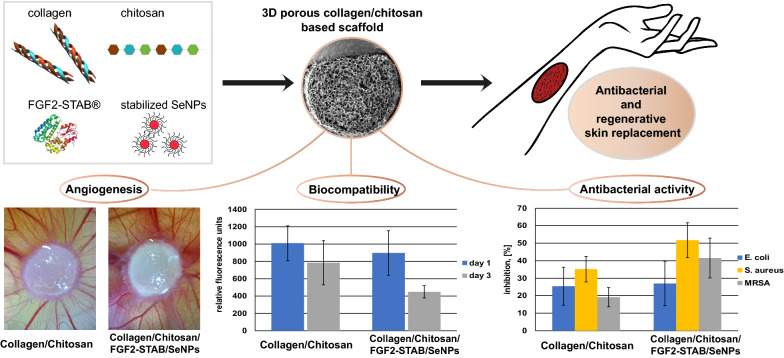

## Background

Hydrogels have become increasingly studied as matrices for tissue engineering [25]. Naturally derived hydrogel-forming polymers have frequently been used in tissue engineering applications because they are either components of, or have macromolecular properties, similar to the natural extracellular matrix (ECM) [10]. The disadvantage of natural material hydrogels is its poor antimicrobial activity, making it a good substrate for bacterial growth and leading to severe infections and inflammation [16]. This characteristic has led to the need to modify natural material hydrogels to incorporate antibacterial properties to prevent these undesirable side reactions.

Nanomaterials have been shown to be complementary to antibiotics as they have antimicrobial properties which are highly promising as they can combat multidrug-resistant mutants and biofilms [6, 32, 56]. Metal and metal oxide nanoparticles, well known for their highly potent antibacterial effects [27] include silver (Ag), iron oxide (Fe_3_O_4_), titanium oxide (TiO_2_), copper oxide (CuO), and zinc oxide (ZnO).

Selenium nanoparticles (SeNPs) also possess antibacterial, antiviral, antioxidant, and anticancer properties, suggesting they could be suitable as therapeutic candidates to combat infectious diseases [26, 45, 48, 51]. Selenium (Se) is a trace element naturally found in the body in contrast to other nanoparticle materials such as Ag, Cu, or TiO_2_. It is a constituent of selenoproteins, which are important for antioxidant defence systems, thyroid hormone metabolism, and redox control of cell reactions [5, 31]. The amount of Se in the body ranges from 10 to 20 mg in adults and the recommended daily dietary allowance is 55 μg per day with an upper tolerable limit of 400 μg [28]. This supports the suggestion that Se is a unique material with strong potential in biomedical applications.

Studies have demonstrated that elemental selenium nanoparticles have antibacterial effects against gram-positive bacteria *Staphylococcus aureus* [32, 34, 45, 49], Methicillin-Resistant *Staphylococcus aureus* (MRSA) [15, 36] and gram-negative bacteria (*Escherichia coli* [36, 52], *Pseudomonas aeruginosa* [4, 17], *Proteus mirabilis* [38], *Klebsiella pneumoniae*, *Acinetobacter baumannii* [17]). Moreover, SeNPs are also effective against other microorganisms such as yeast (*Candida albicans* [12]) and fungi (*Trichophyton rubrum* [54]).

In more detail, Tran & Webster [45] reported that *S. aureus* bacteria growth was inhibited by SeNPs of 40–60 nm in the concentration range 7.8–31 μg/ml and the SeNPs killed approximately 40% of *S. aureus* bacteria, a pathogen which is often a cause of infection in vascular grafts [45]. Antimicrobial activity of SeNPs was also confirmed in the work of Hariharan et al. [13], Chudobova et al*.* [19] and Cihalova et al. [6], where the concentration of SeNPs 300 μM (23.7 μg/ml) and 50–100 nm in diameter caused total *S. aureus* growth inhibition [6, 13, 19]. Another study of selenium nanoparticles showed strong growth inhibition against *S. aureus* at a concentration as low as 1 μg/ml [44].

The antimicrobial activity of selenium nanoparticles is mainly due to the production of reactive oxygen species (known as ROS) leading to disruption of the phospholipid bilayer [11, 29]. In addition to the high antibacterial activity of SeNPs, they can also be easily prepared for use in biomedical applications [34].

As for Se nanoparticles, there are some seemingly contradictory reports regarding the potential toxicity of Se [15, 42] and the biocompatibility of Se, as an antioxidant nanomaterial [1, 23]. Concerning another studies, elemental Se is considered the least toxic form of Se [39, 55], therefore SeNPs could have significant potential to be used as an antimicrobial agent with low toxicity [40]. However, the antibacterial effects of SeNPs are not fully understood and their potential toxicity towards human tissues has been further investigated [49, 53]. Tran et al. [44] aimed to evaluate the cytotoxicity of Se nanoparticles and found that SeNPs showed low toxicity toward fibroblasts which remained more than 70% viable at Se concentrations as high as 128 μg/ml. The nanoparticles also exhibited very low haemolysis with only 18% of maximal lysis observed at a Se concentration of 128 μg/ml [44]. Another potential limitation affecting the clinical application of metal/metalloid nanoparticles is the ability of some nanostructured materials to stimulate processes that trigger unwanted side-effects such as hypersensitivity reactions, autoimmune diseases and inflammatory responses [7, 18]. SeNPs have been used in many treatments of disease conditions including cancer, diabetes, inflammatory disorders, liver fibrosis, and drug induced toxicities [2, 22].

This study aims to extend our previous work on collagen-based scaffolds modified with chitosan [3] and enriched with thermostable fibroblast growth factor 2 (FGF2-STAB^®^) [9]. Biodegradable collagen/chitosan/FGF2-STAB^®^ scaffolds revealed promising results in cell culture experiments and have displayed high suitability and biocompatibility to be used as a transferable scaffold for tissue reconstruction. Firstly, this work aims to study the concentrations of FGF2-STAB^®^ used for scaffold preparation and its impact on fibroblasts' metabolic activity and angiogenesis. Secondly, this work aims to determine the antibacterial properties of scaffolds enriched with SeNPs to study their potential for biomedical and clinical applications in the fields of tissue engineering and regenerative medicine. The combination of FGF2-STAB^®^ and SeNPs was also studied in vitro to test both metabolic activity and antimicrobial activity, which is crucial information for the proper functioning of the scaffold.

The final aim of this study is to develop a complex tissue replacement that is based on naturally derived materials, possesses antibacterial properties, and has the ability to accelerate wound healing.

## Methods

### Materials and chemicals

Bovine type I collagen, 8% aqueous solution (Collado, Czech Republic), low viscosity Chitosan from crab shells, *N*-(3-Dimethylaminopropyl)-*N*´-ethylcarbodiimide hydrochloride (EDC) and *N*-hydroxysuccinimide (NHS) (all from Sigma-Aldrich, Germany) were used as received without further purification. Hyperstable purified FGF2-STAB^®^ growth factor (having thermal stability 20 days at 37 °C was purchased from Enantis, Czech Republic) with concentration 2 µg/ml was used as received.

### Preparation of samples

#### Selenium nanoparticles preparation

SeNPs were synthesized according to the procedure published in our previous study [6, 14]. Complex of selenium nanoparticles stabilized with chitosan were prepared by a reduction of Na_2_SeO_3_ with mercaptopropionic acid in the presence of chitosan. High purity chemicals were used and nanoparticles were purified by dialysis. Size of the nanoparticles evaluated by ZetaSizer (NANO-ZS, Malvern Instruments) differs from 55 to 500 nm with the most abundant diameter between 100 and 200 nm (see SeNPs morphology in Fig. 10h). The content of selenium in SeNPs was checked by atomic absorption spectrometer 280Z (Agilent Technologies, Santa Clara, CA, USA) with electrothermal atomization and selenium ultrasensitive hollow cathode lamp after microwave digestion. The spectrometer operated at 196.0 nm resonance line with spectral bandwidth of 1.0 nm. The determined Se content was 663 ± 14 ppm.

#### Collagen/Chitosan sponges

Collagen/Chitosan sponges were prepared by freeze-drying from 1% polymer aqueous solution and followed by cross-linking with the mixture of EDC/NHS according to our previous work [41]. Briefly, 0.5 wt % collagen mixtures were prepared from lyophilized 100% collagen in ultrapure water type II (Millipore filtration system according to ISO 3696). Chitosan was used as an additive in the ratio of 1:1 (wt/wt). The mixtures were homogenized and freeze-dried. Samples were primary freeze-dried in Martin Christ Epsilon 2-10D lyophilizator at − 35 °C under 1 mBar for 15 h followed by a secondary drying process at 25 °C under 0.01 mBar until decreasing Δp up to 10%. Samples were additionally cross-linked with known carbodiimide system (EDC/NHS in molar ratio 2/1) and after removal of byproducts followed by freeze-drying again.

#### Collagen/Chitosan sponges enriched by FGF2-STAB^®^ and SeNPs

**Fig. 1:**
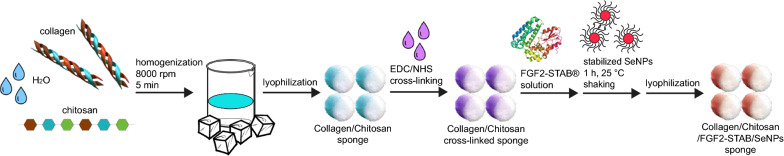
Scheme of Collagen/Chitosan sponge fabrication followed by FGF2-STAB^®^ and SeNPs enrichment procedure

Scaffold enrichment with FGF2-STAB^®^ water solution and/or SeNPs was performed after lyophilization of biopolymer mixture and cross-linking process. FGF2-STAB^®^ and/or SeNPs at certain concentrations were poured onto the cross-linked sponge, left for 1 h shaking at 25 °C and re-lyophilized again (Fig. 1).

### Normal human dermal fibroblasts isolation and culture

Normal human dermal fibroblasts (NHDF) were isolated from skin residues after plastic surgery interventions after patients’ informed consent by digestion-migration method, or from human skin which was collected as waste tissue from routine surgery. The skin samples were removed during cosmetic plastic surgery at the Department of Plastic Surgery of the University Hospital in Pilsen (Czech Republic), under informed agreement of the donors and after approval by the ethical committee of the University Hospital in Pilsen (Pilsen, Czech Republic). The guidelines specified within the Declaration of Helsinki were followed. For experiments conducted in the UK written informed consent was collected from donors according to a protocol approved by the UK’s National Health Service (NHS) research ethics committee (ref: 15/YH/0177).

Immediately after skin biopsy, samples were immersed into physiological solution and transported into the cell culture lab for immediate isolation. Samples were washed by Hank’s balanced salt solution (HBSS) (Sigma Aldrich) containing penicillin (100 U/ml)/streptomycin (0.1 mg/ml) (Biochrom, United Kingdom) and gentamicin (50 μg/ml) (Biochrom). The samples were cut into 3 mm^2^ pieces and digested overnight at 37 °C in Petri dish (Techno Plastic Products, Trasadingen, Switzerland) in HBSS containing collagenase type I (100 U/ml, Thermo Fisher Scientific, USA). Next day, the suspension containing digested tissue was intensively shaken with vortex for 30 s, filtered through a 100 µm nylon cell strainer (Falcon™, Thermo Fisher Scientific) and the cell suspension was transferred into a 75 cm^2^ cultivation flask (Techno Plastic Products) containing 10 ml of culture medium, which was composed of low glucose Dulbecco’s Modified Eagle’s Medium (DMEM) (Thermo Fisher Scientific), 10% heat-inactivated fetal bovine serum (FBS) (Thermo Fisher Scientific), penicillin (100 U/ml)/streptomycin (0.1 mg/ml) (Biochrom), 0.5% l-glutamin (Biosera, France) and 1.0% non-essential amino acids (Biosera). NHDF were cultured at 37 °C, 5% CO_2_ up to 80% confluence and passaged. NHDF in the 3rd–5th passage were used for all experiments.

### Biocompatibility using normal human dermal fibroblasts

To investigate biocompatibility via fibroblast’s viability of 3D scaffolds and solutions with various concentrations of SeNPs and FGF2-STAB^®^, normal human dermal fibroblasts were used. The fibroblasts were grown in Dubecco’s modified eagle medium (DMEM D6546-500 ml Sigma-Aldrich) supplemented with 10% inactivated fetal bovine serum (FBS), 1% penicillin–streptomycin and 1% l-glutamine in a standard cell culture incubator (37 °C, humidified, 5% CO_2_, 20% O_2_ environment, nonshaking).

The solutions with various concentrations of SeNPs and FGF2-STAB^®^ were incubated in a standard cell culture incubator for 72 h for the proliferation assays, the 3D scaffold samples were incubated for 3, 7, and 10 days. To ascertain the IC50 value, the ultra-high concentration of selenium nanoparticles was used.

The alamarBlue (AB) assay was carried out according to the manufacturer’s instructions. Briefly, the control medium was removed; the cells were rinsed with phosphate-buffered saline (PBS) and 0.5 ml for 2D and 1.5 ml for the 3D experiment of an AB solution (5% [v/v] solution of alamarBlue™ Dye, ThermoFisher Scientific) prepared in the fresh medium were added to each well. Following 4 h incubation for 2D and 3 h for the 3D experiment, AB absorbance was quantified at the wavelength of 562 nm using a BIO-TEK^®^ ELx800 microplate reader. The results were averaged over 3 different independent experiments (n = 3, each conducted with a one-week interval) with 3 replicates per experiment (3 × 6 well plates), each replicate being prepared from different T75 flasks to take into account the biological variability. Finally, for each plate, the reading was also done in triplicate (values obtained from 3 different wells averaged) to include the technical variability due to the efficiency of AB assay, the sensitivity of the plate reader, or simply related to the sample preparation. For each experiment, wells containing only the AB solution without cells were also prepared and incubated for 3 or 4 h. The absorbance measured in those was used as a background and subtracted.

#### Biocompatibility of scaffolds

200.000 NHDF cells in 200 µl of the diluted cell suspension were applied directly on to the 3D scaffolds and after 2 h added 1.3 ml of media into each well with the scaffold*.* After 3, 7 and 10 days, the absorbance of AB was measured using a microplate reader at a wavelength of 562 nm.

#### Biocompatibility of SeNPs and FGF2-STAB^®^

To each well of a 24-well microtiter plate, 20.000 NHDF cells in 500 µl of the diluted cell suspension was applied. Different nanoparticle concentrations (0, 1, 5, 10, 50 and 100 µg/ml) prepared in maintenance media were added. The media with zero nanoparticle concentration were considered for positive control.

After 24 and 72 h, the absorbance was measured using a microplate reader at a wavelength of 562 nm.

The same experiment procedure was carried out with different FGF2-STAB^®^ concentrations (0.01, 0.05, 0.10, 0.50, 1.00 µg/ml), and the same experiment procedure was carried out also for various combinations of concentrations for both agents—SeNPs (0, 1, 5, 10, 50 and 100 µg/ml) and FGF2-STAB^®^ (0.01, 0.05, 0.10, 0.50, 1.00 µg/ml).

### Ex ovo CAM assay

On the day of arrival (day 0), 36 fertilized chicken eggs were received from Henry Steward & Co. (UK) and were placed in an R-Com King Suro 20 digital egg incubator at 37.5 °C to allow the embryos to develop. On day 3, embryos were removed from their eggshells and transferred into 100 ml weighing boats containing 2 ml PBS with a 1% addition of penicillin–streptomycin (PS). The weighing boats were then placed in Petri dishes containing 12 ml distilled water and lids were placed over the weighing boats to conserve humidity. By removing the embryos from the shells, chick chorioallantoic membranes (CAMs) were exposed, allowing for clearer images to be obtained [8]. The embryos were then transferred to trays in a humidified Binder Classic Line incubator and allowed to develop further at 37 °C. On day 7, a 3D scaffold sample wet by 0.2 ml PBS, was placed onto the surface of each CAM and again allowed to develop in Binder incubator. Images were taken of the samples and surrounding vasculature on days 7 and 10–13, using Motorola USB microscope coupled with Microcapture imaging software. On day 13 the embryos were sacrificed and the scaffold samples (including a small amount of the surrounding CAM) were explanted and fixed by placing in 3.7% formaldehyde for at least 24 h.

To quantify angiogenic properties, the blood vessels growing perpendicularly (within ± 45°) towards the scaffold were ranked, manually counted, and recorded. Analysis of vascular response was based on a total increase in blood vessels growing perpendicularly towards the sample, by subtracting the number of vessels seen on day 7 for a particular sample. All samples were blinded before counting to eliminate the risk of bias. The vasculogenic index corresponds to the number of newly created vessels between days 7 and 10 after fertilization.

### Biocompatibility on primary normal human dermal fibroblasts

#### NHDF seeding into scaffolds

All scaffolds were pre-incubated 1 h before NHDF seeding in 150 μl of culture medium in 96 well plate (Techno Plastic Products, Switzerland) to ensure treminated swelling and volume increase. After NHDF passage and counting in Bürker chamber, culture medium was aspirated from the scaffolds and 20 000 NHDF were seeded on each scaffold in 150 μl of culture medium containing only 0.5% FBS (previously used 10% FBS) to detect the possible effect of FGF2-STAB^®^ in scaffolds. NHDF were cultured for 1 and 3 days followed by metabolic activity assay and by microscopic evaluation. Scaffolds Collagen/Chitosan represented untreated control and scaffolds Collagen/Chitosan enriched by SeNPs and/or by FGF2-STAB^®^ represented tested samples.

#### Metabolic activity assay

Metabolic activity of NHDF was estimated by alamarBlue assay converting blue resazurin to pink resofurin. After 1 or 3 days of NHDF culture in scaffolds, each scaffold was transferred into another well of 96 well plate to prevent signal from NHDF attached to bottoms of wells. Afterwards, 100 μl of alamarBlue solution (ThermoFisher Scientific) 10 × diluted in the culture medium containing 0.5% FBS was added to each scaffold and NHDF were incubated for 2 h at 37 °C, 5% CO_2_. Afterwards, 100 μl of culture medium was transferred into a black 96-well test plate (ThermoFisher Scientific) and fluorescence was measured at 530 nm (ex) and 590 nm (em) in a microplate reader (Synergy HT, Biotek, USA). Results are expressed as mean ± SD from 3 independent NHDF donors.

#### Live/dead assay

Alive NHDF in the scaffolds were visualized by vital intracellular stain calcein-AM and dead NHDF in the scaffolds were visualized by propidium iodide (PI). After 1 or 3 days of NHDF culture in scaffolds, each scaffold was transferred into another well of 96 well plate to prevent signal from NHDF attached to the bottoms of wells. 150 μl of culture medium with 0.5% FBS and containing calcein-AM (1 μg/ml), PI (1 μg/ml) and nuclear stain Hoechst 33,342 (1 μg/ml) (all ThermoFisher Scientific) were added to each scaffolds and NHDF were incubated for 30 min at 37 °C, 5% CO_2_. Afterwards, each scaffold was washed by PBS and transferred into Petri dish with a thin glass bottom covered by fresh culture medium to perform microscopic analysis using Olympus IX83. Fluorescent pictures were taken using objective 4x (NA0.15) at 488 nm for calcein, at 561 nm for PI, and at 405 nm for Hoechst 33,342. Images were standardized for publication in ImageJ (NIH, Bethesda, Maryland, USA).

### Antibacterial properties

The antibacterial properties of the prepared samples were tested on different bacterial strains from Czech Collection of Microorganisms (Brno, Czech Republic), which represented by both gram-positive (*Staphylococcus aureus* NCTC 8511 and MRSA CCM 7110) and gram-negative (*Escherichia coli* NCTC 13216),bacteria. Bacterial cultures were cultured on blood agar plates overnight at 37 °C.

Bacterial cultures were diluted in PBS to the optical density (600 nm) corresponding to 0.5 McFarland turbidity. The diluted bacterial suspension was further diluted 1:100 in Mueller–Hinton broth (Oxoid, UK) to the cell density ∼ 1 × 10^6^ CFU/ml (where CFU is the colony forming unit). Each test tube contained 1 ml of this diluted culture, followed by a piece of sample (d = 8.37 mm, h = 1.5 mm). Samples were incubated for 24 h at 37 °C with continuous rotation (Rotator Multi Bio RS-24, Biosan, Latvia) and the optical density reads (620 nm) were monitored at predetermined time intervals (0, 7, 24 h) using Multiskan EX (Thermo Fisher Scientific, Bremen, Germany).

### Morphology

The morphology and microstructure of lyophilized collagen scaffolds were studied using scanning electron microscopy (SEM), Tescan, Lyra3 XM (Tescan, Brno, Czech Republic). For better resolution, the samples were coated with the 20 nm of gold layer (except SeNPs observation). All observations were made in the secondary electron emission mode at 5 kV acceleration voltage.

Pore size of the scaffolds was characterized from the SEM visualization using the image analyses program ImageJ. Average pore size was calculated from 100 measured values from images collected with magnification 274×.

### Statistical analysis

All values are expressed as mean ± SD and came from at least 3 independent repetitions. Data normality was checked by Shapiro–Wilk test. Non-parametric Mann–Whitney test with Bonferroni correction was applied for in vitro biocompatibility data analysis since the assumption of normality was infringed and Kruskal–Wallis test H. Parametric One-way ANOVA with Bonferroni correction was applied for angiogenic properties evaluation and the level of significance was set at 0.05.

## Results and discussion

### Biocompatibility using human dermal fibroblasts cell line

#### Biocompatibility of scaffolds

According to our previous studies [6, 9, 19] the FGF2-STAB^®^ concentration of 2 µg/ml and the SeNPs concentrations of 2, 10 and 20 µg/ml were selected for Collagen/Chitosan scaffold preparation. Table 1 summarizes prepared Collagen/Chitosan sponges for characterization.

**Table 1:** Prepared Collagen/Chitosan scaffolds

Scaffold	c (FGF2-STAB^®^), (µg/ml)	c (SeNPs), (µg/ml)
Collagen/Chitosan	0	0
Collagen/Chitosan/SeNPs2	0	2
Collagen/Chitosan/SeNPs10	0	10
Collagen/Chitosan/SeNPs20	0	20
Collagen/Chitosan/ FGF2-STAB2	2	0
Collagen/Chitosan/ FGF2-STAB2/SeNPs2	2	2
Collagen/Chitosan/ FGF2-STAB2/SeNPs10	2	10
Collagen/Chitosan/ FGF2-STAB2/SeNPs20	2	20

**Fig. 2:**
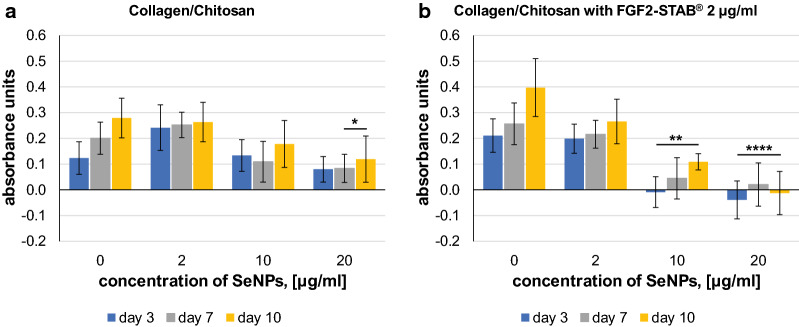
NHDF metabolic activity represented in absorbance units in dependence on increasing SeNPs concentration for Collagen/Chitosan scaffolds after 3, 7 and 10 days of incubation **a** without FGF2-STAB^®^ and **b** with 2 µg/ml of FGF2-STAB^®^. Data represent the mean ± SD of minimum 6 biologically independent experiments with technical triplicate each. P-value below 0.05 (*) are considered statistically significant (Kruskal–Wallis test H)

Metabolic activity of Normal Human Dermal Fibroblasts (NHDF) was estimated by alamarBlue assay on all prepared scaffolds (Fig. 2).

Scaffolds enriched with FGF2-STAB^®^ displayed better NHDF metabolic activity compared to Collagen/Chitosan scaffolds without FGF2-STAB^®^, however the results were not statistically singnificant. Contrary, scaffolds enriched with both FGF2-STAB^®^ and SeNPs at higher concentrations (Collagen/Chitosan/FGF2-STAB2/SeNPs10, Collagen/Chitosan/FGF2-STAB2/SeNPs20), exhibited lower metabolic activity compared to scaffolds enriched with SeNPs only (Collagen/Chitosan/SeNPs10, Collagen/Chitosan/SeNPs20). This finding suggested there were interactions between SeNPs and FGF2-STAB^®^ and prompted a more detailed exploration of the synergistic or antagonistic effects of SeNPs with FGF2-STAB^®^.

#### Biocompatibility of SeNPs and FGF2-STAB^®^

A previous study on the cytotoxicity of SeNPs on fibroblasts was conducted, where concentrations as high as 128 µg/ml of SeNPs were considered nontoxic [44]. Based on the Fig. 2 results exhibiting antagonistic effects of SeNPs with FGF2-STAB^®^ at SeNPs concentrations lower than 10 µg/ml, further cytotoxicity studies were carried out on NHDF with extreme concentrations of either SeNPs or FGF2-STAB^®^ in solution (without scaffold).

**Fig. 3:**
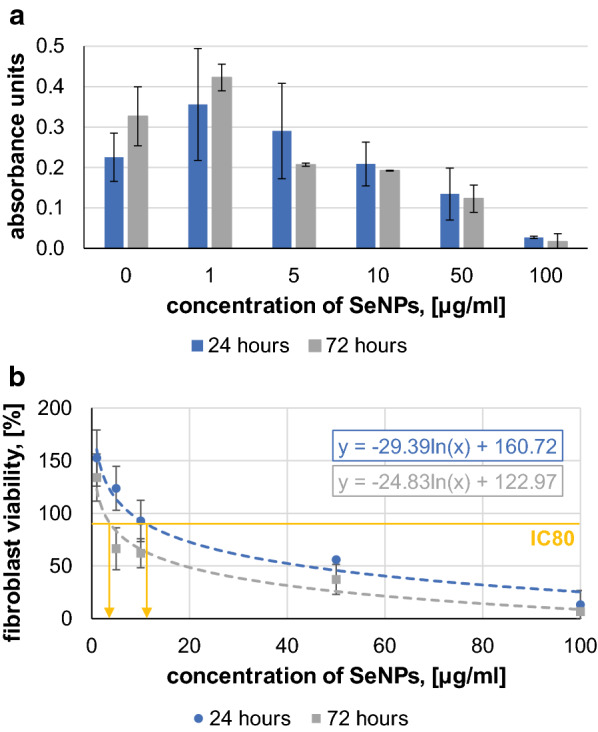
**a** Fibroblast metabolic activity with increasing SeNPs concentration after 24 h and 72 h of incubation, **b** Percentage fibroblast viability relative to the positive control at each day with varying SeNPs concentrations, used to calculate IC50 and IC80

For SeNPs cytotoxicity evaluation, different nanoparticle concentrations (0, 1, 5, 10, 50 and 100 µg/ml) were prepared in maintenance media and were tested (Fig. 3).

According to results from Fig. 3, the concentration of SeNPs at viability 50% and 80% was calculated as IC50 and IC80, respectively (Table 2). The IC50 value indicates the concentration of selenium nanoparticles needed to inhibit a biological process (i.e., fibroblast viability) by half. Although there is no agreed value for tissue replacement, we used the IC80 value (where 80% of fibroblasts remain viable) to determine concentrations suitable for future scaffold development. At this point we can conclude that SeNPs concentrations of 10 and 20 µg/ml previously used in scaffolds induced unacceptable cytotoxicity. According to Table 2, concentration of 5.6 µg/ml of SeNPs are considered as safe for scaffolds applicable in tissue replacement.

**Table 2:** Calculation of IC50 and IC80 of SeNPs on human dermal fibroblasts

Time	Equation	IC50, (µg/ml)	IC80, (µg/ml)
24 h	y = − 29.39ln(x) + 160.72	43.3	15.6
72 h	y = − 24.83ln(x) + 122.97	18.9	5.6

**Fig. 4:**
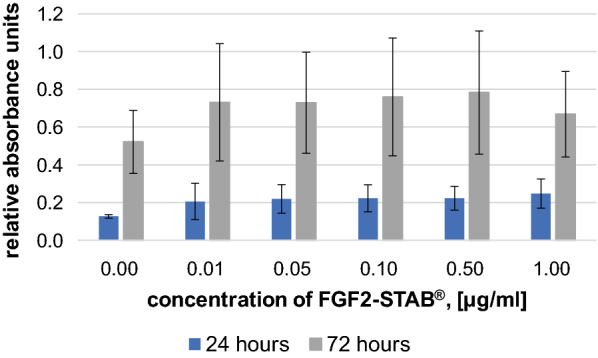
Fibroblast viability with increasing FGF2-STAB^®^ concentrations (0, 0.01, 0.05, 0.10, 0.50, 1.00 µg/ml) after 24 h and 72 h of incubation

The same experimental procedure was then carried out with different FGF2-STAB^®^ concentrations (0, 0.01, 0.05, 0.10, 0.50, 1.00 µg/ml) in solution (Fig. 4). The media with zero FGF2-STAB^®^ concentration were considered for positive control. The addition of FGF2-STAB^®^ into the scaffold seems to have a small positive effect already at very low concentrations of 0.01 µg/ml, but without statistical significance. In our experimental set-up the fibroblast medium contained fetal bovine serum, which contains a large number of nutritional and macromolecular factors essential for cell growth, a potential explanation for why FGF2-STAB^®^ itself did not induce the positive effects expected from previous studies [20].

**Fig. 5:**
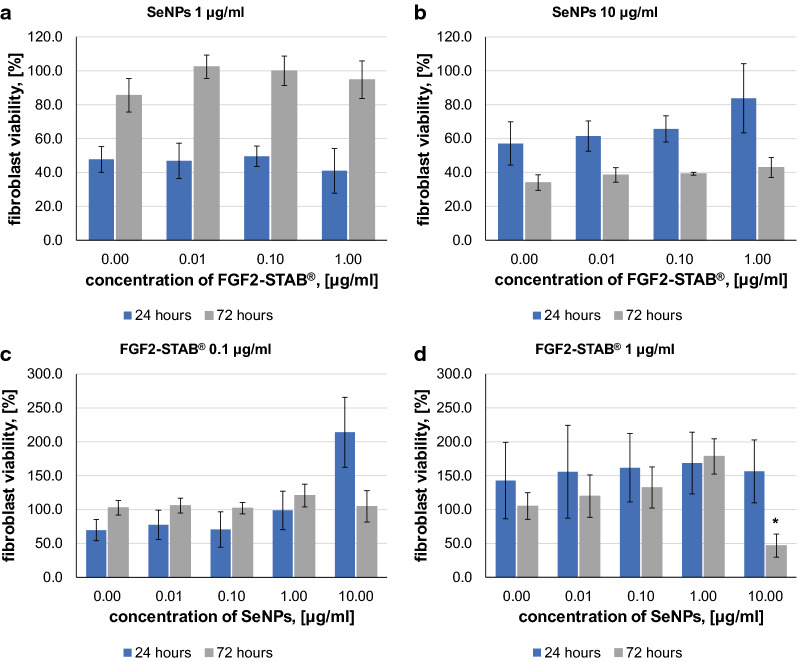
Fibroblast viability relative to positive control at each day **a** for SeNPs concentration of 1 µg/ml with increasing FGF2-STAB^®^ concentrations (0, 0.01, 0.10, 1.00 µg/ml), **b** for SeNPs concentration of 10 µg/ml with increasing FGF2-STAB^®^ concentrations (0, 0.01, 0.10, 1.00 µg/ml), **c** for FGF2-STAB^®^ concentration of 0.1 µg/ml in with increasing SeNPs concentrations (0.0, 0.01, 0.10, 1.00, 10.00 µg/ml), **d** for FGF2-STAB^®^ concentration of 1 µg/ml with increasing SeNPs concentrations (0.0, 0.01, 0.10, 1.00, 10.00 µg/ml) after 24 h and 72 h of incubation. Data represent the mean ± SD of 3 biologically independent experiments with technical triplicate each. P-value below 0.05 (*) are considered statistically significant (Kruskal–Wallis test H)

In order to understand how the two additives interacted, an experiment was conducted with different concentrations of SeNPs and FGF2-STAB^®^ in combination (Fig. 5a–d).

Figure 5a shows no effect of SeNPs at a concentration of 1 µg/ml on the metabolic activity of fibroblasts with increasing concentration of FGF2-STAB^®^. Figure 5b demonstrates that 10 µg/ml of SeNPs leads to a reduction in cell metabolic activity of fibroblasts, which confirms the previous findings shown in Table 2 (IC80 value calculation).

In general, from the results presented in Fig. 5a, b we can conclude, that selenium nanoparticles do not have a significant effect on viability at lower concentrations (up to 1 µg/ml of SeNPs), but have a negative effect on viability at higher concentrations (above 10 µg/ml of SeNPs), especially after 72 h of incubation, in combinafion with and FGF2-STAB^®^.

The results in Fig. 5c, d show the synergistic effect of SeNPs with FGF2-STAB^®^ in combination. Based on the data shown here, the combination of 1 µg/ml FGF2-STAB^®^ and 1 µg/ml of SeNPs appears to be the optimal concentration for cell metabolic activity, with an increase after 72 h. However, the 1 µg/ml concentration of FGF2-STAB^®^ with SeNPs at 10 µg/ml concetration exhibited significant decreasing in fibroblast metabolic activity after 72 h (Fig. 5d).

According to Monopoli et al*.* the interaction of NPs with biological media is key in the transport of NPs across the cell membrane. When NPs are exposed to fluids that contain proteins and other biomolecules (e.g. FGF2), part of those biomolecules is immediately adsorbed forming the so-called “protein corona” [30]. The preparation process involves incorporation of SeNPs in the scaffolds firstly and incubation with FGF2-STAB^®^ secondary.

Biomolecules in the environment adsorb strongly to the bare nanoparticle surface, forming a tightly bound layer of biomolecules, the ‘hard’ corona, in immediate contact with the nanoparticle. Other biomolecules, the ‘soft’ corona, have a residual affinity to the nanoparticle–hard-corona complex, but this is much lower, so that molecules are in rapid exchange with the environment. If sufficiently long-lived in the corona, a biomolecule may lead to recognition of the nanoparticle–corona complex as a whole by a cell membrane receptor. The same biomolecule alone can also be recognized by the receptor [30]. The size, shape, and surface characteristics of NPs affect protein adsorption and have the ability to modify the structure of the adsorbed protein molecules which can significantly affect the reactivity of the NP with cells [37]. This may be the reason, why the metabolic activity of fibroblasts for a combination of SeNPs and FGF2-STAB^®^ is lower than that for SeNPs alone. The cell receptor can recognize nanoparticle–corona complex as FGF2-STAB^®^ itself and the nanoparticle inside the cell may have a negative impact. Kalishwaralal et al. reported a study, where the cellular functions of vascular endothelial growth factor (VEGF) have been affected by silver nanoparticles. Silver nanoparticles inhibit VEGF induced endothelial cell migration. When VEGF was added, more endothelial cells migrated when compared with the control. While the significant area of the wound is uncovered in plates treated with 500 nM of silver nanoparticles in the presence and absence of VEGF [21]. This finding is significant because VEGF as well as FGF2-STAB^®^ mediated proliferation and migration has a central role in many pathological conditions like wound healing and chronic inflammation.

At this point, it is important to choose the optimum concentration of additives. According to metabolic activity assays, the best combination of agents is 0.01–1 µg/ml of SeNPs and 0.01–1 µg/ml of FGF2-STAB^®^.

### Angiogenic properties of scaffolds

Concerning tissue regeneration and wound healing, the formation of new blood vessels from the endothelium of the existing vasculature plays a fundamental role. Since these scaffolds contain various bioactive agents, which have the potential to modulate angiogenesis, a CAM assay was possible to test the angiogenic potential in an ex ovo environment.

**Fig. 6:**
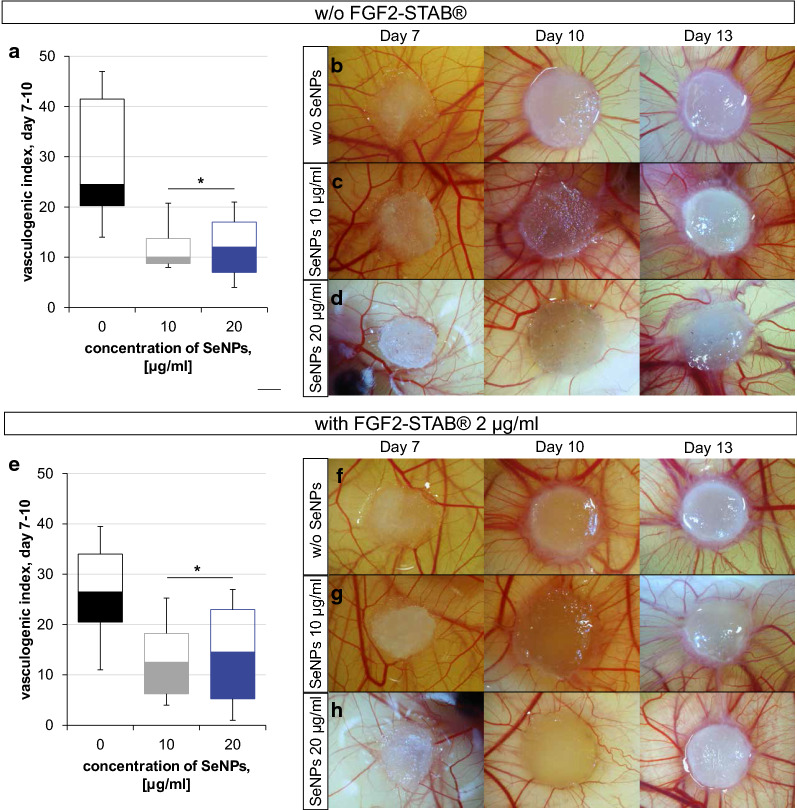
**a** Box plot of vasculogenic index between day 7 and day 10 for Collagen/Chitosan samples without FGF2-STAB®. Collagen/Chitosan samples implanted on CAMs at day 7, 10 and 13 without FGF2-STAB^®^, **b** Collagen/Chitosan, **c** Collagen/Chitosan/SeNPs10, **d** Collagen/Chitosan/SeNPs20. **e** Box plot of vasculogenic index between day 7 and day 10 for samples with FGF2-STAB^®^ 2 µg/ml. Collagen/Chitosan samples implanted on CAMs at day 7, 10 and 13 with FGF2-STAB^®^ 2 µg/ml **f** Collagen/Chitosan/FGF2-STAB2, **g** Collagen/Chitosan/FGF2-STAB2/SeNPs10, H) Collagen/Chitosan/FGF2-STAB2/SeNPs20. P-value below 0.05 (*) indicates significant differences between enriched scaffolds and the dedicated control scaffold (Collagen/Chitosan without SeNPs) (One-way ANOVA with Bonferroni correction)

Collagen/Chitosan scaffolds alone or dopped with 10 or 20 µg/ml of SeNPS were compared with those having added 2 µg/ml of FGF2-STAB^®^.The amount of vessels of each sample was evaluated at day 7 and 10 and their difference known as vasculogenic index was compared (Fig. 6a–h). As expected, CAM assay observations confirmed the results presented by in vitro examination. Addition of FGF2-STAB^®^ increased the vasculogenic index slightly, while the addition of high SeNPs concentrations of 10 and 20 µg/ml caused a significant decrease in vasculogenic index in comparison to scaffolds without SeNPs (Fig. 6a, e). Further studies are needed to test the effect of lower concentrations of SeNPs on angiogenesis. All embryos were traced up to day 13 to be sacrificed. Some embryos did not survive until day 13 and died during the experiment especially at later stage due to the nonspecific inflammatory reaction. The highest percentage of embryos that survived until day 13 ranged from 85 to 92% for samples containing high amounts of SeNPs (Collagen/Chitosan/FGF2-STAB2/SeNPs20 and Collagen/Chitosan/SeNPs20). Contrary, the lowest value of only 62% exhibited sample without selenium containing FGF2-STAB^®^ (Collagen/Chitosan/FGF2-STAB2). It is assumed, SeNPs could promote the antibacterial effect of penicillin–streptomycin, which is added to the PBS solution to prevent infection and thus premature embryo death. Although SeNPs were present in high concentrations in these samples (20 µg/ml), enrichment with SeNPs showed a positive effect on embryo survival.

### Biocompatibility on primary normal human dermal fibroblasts

Following the results presented above, new scaffolds were prepared for biocompatibility evaluation with lower concentrations of the bioactive agents. According to metabolic activity assays, we selected the combination of agents with 0.5, 1.0 and 5.0 µg/ml of SeNPs and 0 and 1 µg/ml of FGF2-STAB^®^. Table 3 summarizes the prepared Collagen/Chitosan scaffolds for repetition of biocompatibility evaluation.

**Table 3:** Prepared Collagen/Chitosan scaffolds for repetition of biocompatibility evaluation

Scaffold	Label	c(FGF2-STAB®), (µg/ml)	c(SeNPs), (µg/ml)
Collagen/Chitosan	C/Ch	0	0.0
Collagen/Chitosan/SeNPs0.5	C/Ch_Se0.5	0	0.5
Collagen/Chitosan/SeNPs1	C/Ch_Se1	0	1.0
Collagen/Chitosan/SeNPs5	C/Ch_Se5	0	5.0
Collagen/Chitosan/ FGF2-STAB1	C/Ch_FGF1	1	0.0
Collagen/Chitosan/ FGF2-STAB1/SeNPs0.5	C/Ch_FGF1_Se0.5	1	0.5
Collagen/Chitosan/ FGF2-STAB1/SeNPs1	C/Ch_FGF1_Se1	1	1.0
Collagen/Chitosan/ FGF2-STAB1/SeNPs5	C/Ch_F1_Se5	1	5.0

**Fig. 7:**
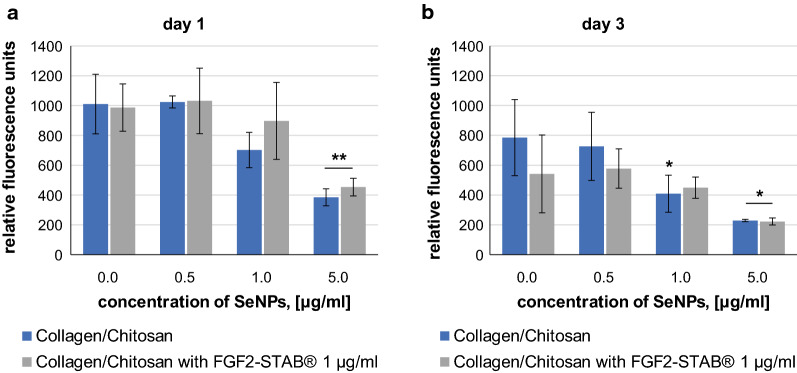
Metabolic activity determined by alamarBlue Assay of normal human dermal fibroblasts (NHDF) cultured in 3D Collagen/Chitosan based scaffolds enriched by SeNPs of different concentrations (0.5, 1.0, 5.0 μg/ml) or enriched by SeNPs of different concentrations (0.5, 1.0, 5.0 μg/ml) and by FGF2-STAB^®^ of concentration 1 μg/ml for 1 day (**a**) and 3 days (**b**). Metabolic activity was assessed by alamarBlue. P-value below 0.05 (*) indicates significant differences between enriched scaffolds and the dedicated control scaffold (Collagen/Chitosan without SeNPs) (Mann–Whitney test with Bonferroni correction)

**Fig. 8:**
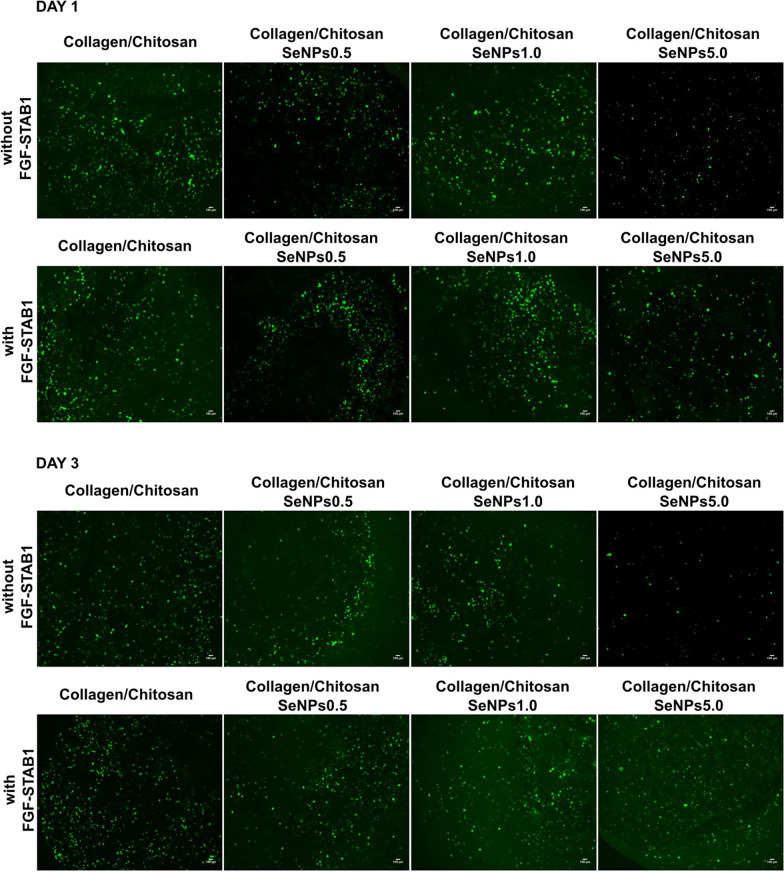
Vital staining of normal human dermal fibroblasts (NHDF) cultured in 3D Collagen/Chitosan based scaffolds enriched by SeNPs of different concentrations (0.5, 1.0, 5.0 μg/ml) or enriched by SeNPs of different concentrations (0.5, 1.0, 5.0 μg/ml) and by FGF2-STAB^®^ of concentration 1 μg/ml for 1 and 3 days. Alive NHDF were stained by calcein-AM (1 μg/ml, 30 min, 37 °C, 5% CO_2_) (green) and dead NHDF in scaffolds were visualized by PI (1 μg/ml, 30 min, 37 °C, 5% CO_2_) (red). Pictures were taken by Olympus IX83 in confocal mode with 4 × objective. Scale bar indicates 100 μm

Metabolic activity and vitality of NHDF cultured for 1 and 3 days in 3D Collagen/Chitosan based scaffolds enriched by SeNPs of different concentrations (0.5, 1.0, 5.0 μg/ml) or enriched by SeNPs of different concentrations (0.5, 1.0, 5.0 μg/ml) and by FGF2-STAB^®^ of concentration 1 μg/ml was assessed by alamarBlue Assay (Fig. 7) and by live/dead staining followed by confocal fluorescent microscopy (Fig. 8), respectively.

At day 1 NHDF exhibited same metabolic activity in Collagen/Chitosan scaffolds, in Collagen/Chitosan scaffolds enriched by SeNPs at concentration 0.5 μg/ml and in Collagen/Chitosan scaffolds enriched by SeNPs at concentration 0.5 μg/ml and by FGF2-STAB^®^ (Fig. 7a). Collagen/Chitosan scaffolds enriched by SeNPs at concentration 1.0 μg/ml exhibited slight cytotoxicity towards NHDF and the addition of FGF2-STAB^®^ reversed this cytotoxic effect. Collagen/Chitosan scaffolds enriched by SeNPs at concentration 5.0 μg/ml without and with FGF2-STAB^®^ exhibited cytotoxicity towards NHDF compared with scaffolds without SeNPs (5.0 μg/ml) (Fig. 7a). At day 3 NHDF exhibited the same metabolic activity in Collagen/Chitosan scaffolds and in Collagen/Chitosan scaffolds enriched by SeNPs at concentration 0.5 μg/ml and in Collagen/Chitosan scaffolds enriched by SeNPs at concentration 0.5 μg/ml and by FGF2-STAB^®^ (Fig. 7b). Collagen/Chitosan scaffolds enriched with SeNPs at concentration 1.0 and 5.0 μg/ml exhibited cytotoxicity towards NHDF compared to Collagen/Chitosan scaffolds without SeNPs. As expected scaffolds enriched with SeNPs at concentration 5.0 μg/ml kept their cytotoxicity even after FGF2-STAB^®^ addition (Fig. 7b).

Vital staining of NHDF showed a similar amount of viable cells (green) on Collagen/Chitosan scaffolds enriched by SeNPs at concentrations 0.5 and 1.0 μg/ml without and with FGF2-STAB^®^. Collagen/Chitosan scaffolds containing SeNPs at concentration 5.0 μg/ml showed lower cell density, however, attached cells remained viable especially after FGF2-STAB^®^ addition, where the vital staining of NHDF is more intensive after 1 and 3 days culture (Fig. 8). Viable NHDF exhibited a typical spindle-like morphology and populated the whole 3-D volume of scaffolds.

### Antibacterial properties of scaffolds

**Fig. 9:**
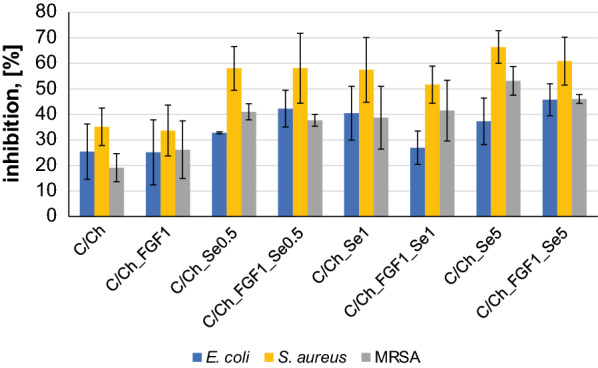
Antibacterial effect of various scaffolds (shown in Table 3) against *E. coli*, *S. aureus* and MRSA. Percentage of bacteria inhibition is relative to the positive control. All measurements were performed in triplicate (mean ± SD for n = 3)

Due to the interactions and incorporation of antibacterial agents into the scaffold structure it is possible that agents, which normally possess antibacterial properties, can lose these properties after addition into scaffolds or when combined with FGF2-STAB^®^. Therefore, experiments were conducted to evaluate the bacterial inhibition of the prepared materials against *E. coli*, *S. aureus* and MRSA (Fig. 9).

According to our previous cytotoxicity evaluation on human dermal fibroblasts, the maximum concentration of SeNPs in the scaffold at 5 µg/ml was established. Previous studies have shown the minimum concentration of SeNPs for antimicrobial effectiveness, 1 µg/ml of SeNPs is needed to inhibit *S. aureus* growth [44].

Antibacterial activity was evaluated by broth method against gram-positive and gram-negative bacteria. Antibacterial ability was tested on Collagen/Chitosan scaffolds, Collagen/Chitosan scaffolds containing SeNPs or FGF2-STAB^®^ and Collagen/Chitosan scaffolds containing both, SeNPs and FGF2-STAB^®^.

Based on the Fig. 9, the increase of SeNPs concentration in Collagen/Chitosan scaffolds resulted in an increase in the antibacterial activity towards all of the tested bacterial strains, especially *S. aureus*. The highest inhibition effect was found for *S. aureus* (51–66%), for MRSA (37–53%) and the lowest activity was monitored in the case of *E. coli* (27–46%). *S. aureus* is the major cause of skin and soft tissue infections, moreover it belongs to the most common pathogen found at surgical site infections [43]*.* In addition, up to half of *S. aureus* clinical isolates are identified as methicillin-resistant [24]. The scaffolds without SeNPs also reduce bacterial growth (inhibition 19–35%), which is likely due to the well-known antibacterial properties of chitosan [47].

Comparing Collagen/Chitosan samples with or without FGF2-STAB^®^ showed that this growth factor had no noticeable antibacterial effects.

It has been noticed that the effect of selenium nanoparticles can significantly differ in gram-positive and gram-negative bacteria. Authors mentioned that the antimicrobial effects of SeNPs on bacterial cultures of *S. aureus* and MRSA (gram-positive bacteria) were enhanced with increasing concentrations. In contrast, the effects on gram-negative bacteria *E. coli* were observed only at the high concentration of 300 µM (23.7 μg/ml) of SeNPs [36, 44].

According to the antibacterial results shown in this study, we can confirm that SeNPs are able to enhance the scaffold’s antibacterial properties towards *S. aureus* and MRSA at concentrations between 0.5 µg/ml and 5 µg/ml. In agreement with the above mentioned studies, here SeNPs provided as well as lower inhibitory effect against gram-negative *E. coli*, however, some inhibition was still observed.

### The effect of additives on the scaffold microstructure

**Fig. 10:**
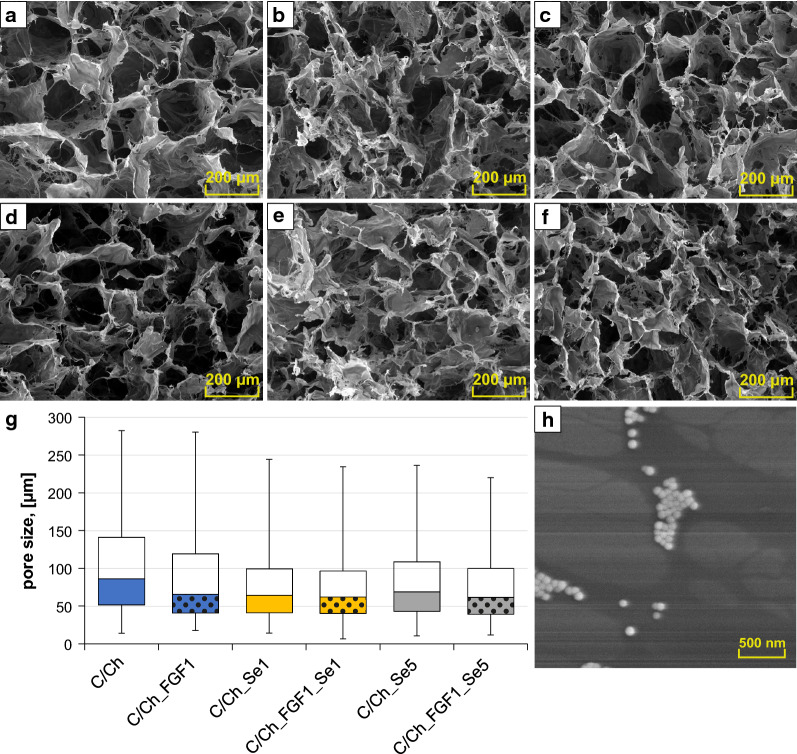
SEM images of all prepared crosslinked collagen sponges with various additives and pore size evaluation. Crosslinked scaffolds represent **a** Collagen/Chitosan, **b** Collagen/Chitosan/SeNPs1, **c** Collagen/Chitosan/SeNPs5. Crosslinked scaffolds enriched by FGF2-STAB^®^ of 1 µg/ml represent **d** Collagen/Chitosan/FGF2-STAB1, **e** Collagen/Chitosan/FGF2-STAB1/SeNPs1, **f** Collagen/Chitosan/FGF2-STAB1/SeNPs5. **g** Pore size box plot for each scaffold. **h** SEM image of the selenium nanoparticles stabilized with chitosan

Different morphologies of prepared Collagen/Chitosan-based 3D scaffolds modified by both FGF2-STAB^®^ and SeNPs are shown as SEM images in Fig. 10a–f. A homogenous network was formed by freeze-drying procedure, where the interconnected 3D porous structure within the samples was created. The additives significantly changed the microstructure visually. Collagen/Chitosan sample without any enrichment showed honeycomb-like structure. The addition of solutions with SeNPs remodeled defined honeycomb structure into the more fibrous structure. Remodeling of the structure is mainly caused by the addition of SeNPs to the scaffolds while the addition of FGF2-STAB^®^ only affected the structure slightly.

Figure 10g represents the pore size box plot of the prepared scaffolds, where the range between 25 and 75th percentile of scaffolds moves from 39 to 141 μm in all samples (without statistical significance), which makes them suitable for cell growth. The Collagen/Chitosan/FGF2-STAB1/SeNPs5 scaffold exhibited the smallest pores of about 62 μm in the median value. In comparison to the Collagen/Chitosan scaffold, all scaffolds enriched with FGF2-STAB^®^ and/or SeNPs showed slightly smaller pore size values. Figure 10h displays SeNPs morphology with scale bar of 500 nm.

### Workflow summary

Although the research builds on our previous work, various samples had to be prepared and analysed to determine the correct concentration of FGF2-STAB^®^ and SeNPs in the scaffolds. For clarity, see the table showing the workflow scheme (Table 4).

**Table 4:** Workflow scheme

No	Material	c (SeNPs) (µg/ml)	c (FGF2-STAB^®^) (µg/ml)	Analysis	Summary
1	Collagen/Chitosan scaffold	0; 2; 10; 20	0; 2	AB assay CAM assay	Antagonistic effect of SeNPs and FGF2-STAB^®^ at high concentrations of SeNPs (10 and 20 µg/ml)
2	Solution	0; 1; 5; 10; 50; 100	/	AB assay IC80 calculation	IC80 is 5.6 µg/ml of SeNPs for HDF
3	Solution	/	0; 0.01; 0.05; 0.1; 0.5; 1	AB assay	Positive effect on fibroblast metabolic activity at a concentration as low as 0.01 µg/ml
4	Solution	1; 10	0; 0.01; 0.1; 1	AB assay	Synergistic effect of SeNPs and FGF2-STAB^®^ was confirmed in solution; the best combination of agents is 0.01–1 µg/ml of SeNPs and 0.01–1 µg/ml of FGF2-STAB^®^
		0; 0.01; 0.1; 1; 10	0.1; 1		
5	Collagen/Chitosan scaffold	0; 0.5; 1; 5	0; 1	AB assay, Life/dead assay Broth method Morphology and pore size analysis	Optimal concentration for Collagen/Chitosan scaffold enrichment is 0.5 µg/ml of SeNPs and 1 µg/ml of FGF2-STAB^®^

In the beginning, scaffolds were prepared with concentrations of FGF2-STAB^®^ and SeNPs that are based on literature (No. 1). The AB assay and the ex ovo CAM assay were performed that indicated an negative effect of SeNPs and FGF2-STAB^®^ at high concentrations of SeNPs (10 and 20 µg/ml). Based on this finding, solutions of FGF2-STAB^®^, SeNPs, and their combinations at different concentrations were tested in more detail in vitro (No. 2, 3, 4). For SeNPs, the IC80 value was calculated based on the AB assay which is 5.6 µg/ml of SeNPs for NHDF (No. 2). From the analysis carried out in No. 3, a positive effect on fibroblast metabolic activity at a concentration as low as 0.01 µg/ml was proved, but no significant effect of the FGF2-STAB^®^ concentration in the range 0.01–1.00 µg/ml. Various combinations of FGF2-STAB^®^ and SeNPs in solution were prepared for another metabolic activity evaluation (No. 4). Here a synergistic effect of SeNPs and FGF2-STAB^®^ was observed as in the previous experiments in No. 1. The best combination of agents turned out to be 0.01–1 µg/ml of SeNPs and 0.01–1 µg/ml of FGF2-STAB^®^. Based on previous results, additional scaffolds were prepared to contain adjusted concentrations of FGF2-STAB^®^ and SeNPs and the AB assay analysis was repeated (No. 5). These scaffolds were also subjected to antibacterial tests and imaged by SEM. In conclusion, up to a concentration of 0.5 µg/ml SeNPs, the antibacterial effect was more dominant, because further addition of SeNPs did not increase antibacterial effects in any case even with FGF2-STAB^®^ enrichment or pure Collagen/Chitosan scaffold. Already low addition of SeNPs significantly increased the antibacterial effect. However, the cytotoxic effect began to predominate at concentrations higher than 0.5 µg/ml of SeNPs, which was amplified by the addition of FGF2-STAB^®^.

## Conclusions

In this study, Collagen/Chitosan scaffolds were enriched with selenium nanoparticles (SeNPs) and hyperstable fibroblast growth factor 2 (FGF2-STAB^®^) to develop safe antibacterial and vasculogenic scaffolds for possible aplication as tissue-engineered skin replacement. This study aimed to determine the optimum concentrations of these two biomolecules in combination with Collagen/Chitosan to ensure a safe and effective dose both in terms of cytotoxicity and biocompatibility and in terms of antibacterial effect. This study has shown that Collagen/Chitosan scaffolds enriched with SeNPs combined with FGF2-STAB^®^ must consider the mutual interaction between biomolecules and the toxic effects of higher SeNPs concentrations. Results showed a synergistic effect of SeNPs and FGF2-STAB^®^ and showed the optimal concentrations were 0.5 µg/ml for SeNPs and 1 µg/ml for FGF2-STAB^®^ for Collagen/Chitosan scaffold enrichment. Scaffolds dopped with 0.5 µg/ml SeNPs and 1 µg/ml FGF2-STAB^®^ were shown to support fibroblast attachment and metabolic activity while also displaying antibacterial activity against three bacterials strains (*E. coli*, *S. aureus* and MRSA).

In the following studies, we address Collagen/Chitosan scaffolds that can release SeNPs more slowly, thus limiting the negative effect of SeNPs. Early release of FGF2-STAB^®^ could promote cell proliferation at the beginning of healing, and gradual slow release of SeNPs could prevent infection in the subsequent stages of healing. This study has demonstrated the importance of understanding the interactions between nanoparticles and proteins in scaffold design and why it is necessary to verify these interactions for the safety of tissue replacements intended for use in the body.

## Data Availability

Without restrictions.

## Abbreviations

AB assay: AlamarBlue Assay
CAM assay: Chorioallantoic membrane assay
Collagen/Chitosan: 3D porous Collagen/Chitosan scaffold
DMEM: Dubecco’s modified eagle medium
EC: Escherichia coli
ECM: Extracellular matrix
EDC/NHS: *N*-(3-Dimethylaminopropyl)-*N*´-ethylcarbodiimide hydrochloride/*N*-hydroxysuccinimide
FBS: Fetal bovine serum
FGF2: Fibroblast growth factor 2
HBSS: Hank’s balanced salt solution
IC50: The half-maximal inhibitory concentration
IC80: The 80% inhibitory concentration
MRSA: Methicillin-resistant Staphylococcus aureus
NHDF: Normal human dermal fibroblast
NPs: Nanoparticles
PBS: Phosphate-buffered saline
PI: Propidium iodide
PS: Penicillin–streptomycin
SA: Staphylococcus aureus
Se: Selenium
SEM: Scanning electron microscope
SeNPs: Selenium nanoparticles
VEFG: Vascular endothelial growth factor
